# Eosinophilic Cystitis Presenting as Possible Pediatric Rhabdomyosarcoma in Conventional Imaging Including ^18^F-FDG-PET/CT/MRI—A Rare Case

**DOI:** 10.3390/diagnostics11040672

**Published:** 2021-04-08

**Authors:** Naja Enevold Olsen, Marie Øbro Fosbøl, Jorgen Thorup, Helle Hjorth Johannesen, Lise Borgwardt

**Affiliations:** 1 Department of Clinical Physiology, Nuclear Medicine & PET, Rigshospitalet, Copenhagen University Hospital, Blegdamsvej 9, DK-2100 Copenhagen, Denmark; naja.enevold.olsen@hotmail.com (N.E.O.); marie.oebro.fosboel@regionh.dk (M.Ø.F.); helle.hjorth.johannesen.01@regionh.dk (H.H.J.); 2 Department of Nuclear Medicine & PET Centre, Aarhus University Hospital, Skejby, Palle Juul-Jensens Boulevard 165, DK-8200 Aarhus, Denmark; 3 Department of Pediatric Surgery, Rigshospitalet, Copenhagen University Hospital, Blegdamsvej 9, DK-2100 Copenhagen, Denmark; joergen.mogens.thorup@regionh.dk; 4 Faculty of Health and Medical Sciences, University of Copenhagen, DK-2200 Copenhagen, Denmark

**Keywords:** eosinophilic cystitis, pediatric rhabdomyosarcoma, ^18^F-FDG-PET/CT/MRI, biomarker

## Abstract

Eosinophilic cystitis (EC) is a relatively rare, but benign inflammatory bladder disease compared to that of the malignant pediatric rhabdomyosarcoma (RMS), in which it can be mimicking on initial suspicion. The origin, symptoms and findings of both EC and RMS are still discussed and hence, lead to the challenge in distinguishing them by cystoscopy and several image modalities. We present a case in which cross-sectional imaging modalities including fluorine-18-fluro-2-deoxy-D-glucose (^18^F-FDG)-positron emission tomography (PET) / computed tomography (CT) / magnetic resonance imaging (MRI) (^18^F-FDG-PET/CT/MRI (The imaging modality ^18^F-FDG-PET/CT/MRI referring to two continuous scans scanned on the same ^18^F-FDG-tracer dose for both the whole-body ^18^F-FDG-PET/CT and the regional ^18^F-FDG-PET/MRI of the pelvis.)) raised suspicion of RMS. Hence, the final diagnosis of EC was established by repeated histopathology. It is important to have EC in mind when seeking differential diagnosis of malignant diseases like RMS in order to provide the correct treatment for the patient and highly homogenously increased ^18^F-FDG-uptake should raise the suspicion of EC as a differential diagnosis. Furthermore, ^18^F-FDG-uptake rate is suggested as a future potential biomarker for monitoring of therapeutic response in eosinophilic inflammatory diseases, thus more research on this topic is needed.

**Figure 1:**
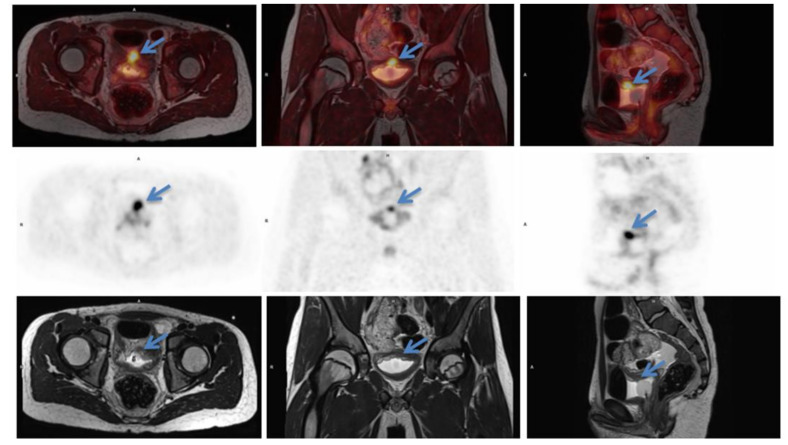
Initial scan: ^18^F-FDG-PET/MRI (T2 weighted) fused images (top row) transaxial, coronal and sagittal view, positron emission tomography (PET) images (middle row) and MRI (T2 weighted) images (bottom row). Suspicious lesion in the bladder wall is marked with blue arrows. 12-year-old previously healthy boy presents with pain in the left flank and dysuria 2 weeks prior to admission. The patient had also noticed a small skin lesion near the external urethral orifice, but no sign of macroscopic hematuria, urine retention or fever. Furthermore, the patient reported episodes of vomiting, intermittent headache, low appetite and a weight loss of 5 kg. The patient had no known allergies besides light seasonal allergic symptoms. At referral to the pediatric surgical department, a minor palpation of the abdomen caused severe pain in the area of the left kidney and less of the right kidney. Blood pressure was 166/102 mmHg (normal range <122/76 mmHg), *p*-creatinine was initial 127 micromol/L increasing to 137 micromol/L (normal range: 39–68 micromol/L) *p*-potassium 4.6 mmol/L increased to 5.0 mmol/L (normal range: 3.3–4.3 mmol/L) and urine-test positive for leucocytes and protein. Ultrasound (US) showed bilateral hydronephrosis with dilated ureters down to the bladder, most prominent on the left side, and reduced cortical thickness of the left kidney. The wall of the bladder was thickened with a prominence in the bottom. Computed tomography (CT) of the abdomen and pelvis confirmed the US findings. Cystoscopy (CS) revealed what could represent a polypoid rhabdomyosarcoma-alike tumor in the trigonum area mostly towards the right side. Multiple biopsies were taken, a double JJ-stent placed on the left side, a nephrostomy on the right side and a catheter. Due to suspicion of malignant disease a whole-body ^18^F-FDG-PET/CT combined with a ^18^F-FDG-PET/MRI of the pelvis was performed. ^18^F-FDG-PET/CT/MRI showed high uptake (SUVmax 9.9) in the top of the bladder, in the trigonum area and more inhomogeneous uptake posterior and in the ureters (Figure 1). Additionally, focal ^18^F-FDG-uptake in the liver hilum was also found and could represent malignant lymph nodes (Figure 2). This supported the initial tentative diagnosis of malignant disease. Renography showed split function right 76 %/left 24 % with severe delay on the left side and normal clearance. However, the final results of the biopsies from the urinary bladder revealed histological inflammation and eosinophilic cystitis. To confirm the benign findings, despite the malignant suspicion on CS and several imaging modalities, CS was repeated and biopsies were obtained again. US of the abdomen, like ^18^F-FDG-PET/CT/MRI, found two small lymph nodes in the liver hilum, but neither of them suspicious of malignancy on US and they could not be biopsied. Meanwhile, tests for concurrent parasitic infection were negative. The second set of biopsies confirmed the diagnosis of eosinophilic cystitis and thus, the patient was treated with high-dose Prednisolone. The increased ^18^F-FDG-uptake of the lymph nodes in the liver hilum, seen on the primary ^18^F-FDG-PET/CT/MRI-scan, were then interpreted as inflammatory. After 3 months of therapy, follow-up CS and ^18^F-FDG-PET/CT significantly showed the benefit of the treatment and now complete remission to normalization (Figure 3 and Figure 4), although the renography showed split function right 71%/left 29% with normalization of the earlier delay on the left side. To our knowledge, there is only one previously published case report describing eosinophilic cystitis (EC) mimicking pediatric rhabdomyosarcoma (RMS) with high uptake on ^18^F-FDG-PET and MRI, and thickening of the bladder wall on US, CS and CT, and our studies are furthermore performed in hybrid scanners as ^18^F-FDG-PET/CT/MRI [1]. The presenting symptoms of EC are hematuria, urine retention, dysuria, suprapubic pain and some have constitutional symptoms, which may increase the suspicion of a malignant disease [2]. Boys are more frequently affected than girls and approximately 30% of patients have a history of other allergic manifestations [3,4], however, this proportion is similar to the general prevalence of allergic conditions in the pediatric population. In conclusion, EC should be kept in mind regarding a differential diagnosis to RMS. This especially when symptoms, findings and imaging modalities can refer to both EC and RMS, but only histopathology can determine the final diagnosis and, in this case, biopsy was repeated in order to rule out malignancy. The uptake of ^18^F-FDG is higher and more homogeneous than most RMS, which is why EC should be kept in mind when this is seen. Moreover, studies are suggesting that ^18^F-FDG-uptake rate could be a potential future biomarker for therapeutic response in eosinophilic inflammation of the airway walls for non-invasive monitoring of asthma, raising the awareness whether it could also apply for monitoring of the therapeutic response and assisting the diagnosis in EC as well. Thus, further research within this field is needed [5].

**Figure 2:**
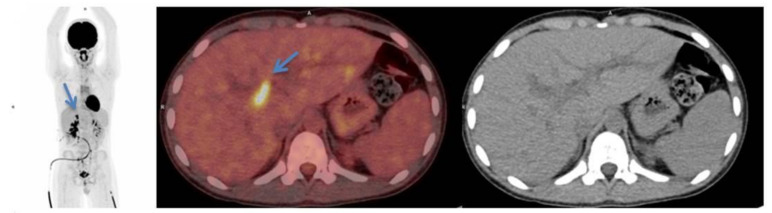
Initial scan: ^18^F-FDG-PET/CT maximal intensity projection (**left**), transaxial fused PET/CT image (**middle**) and CT image (**right**) of the ^18^F-FDG-avid lymph nodes in the liver hilum (blue arrows).

**Figure 3:**
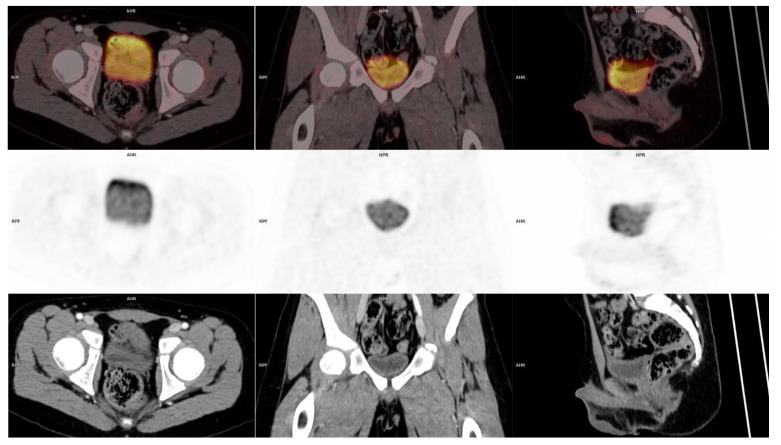
Follow-up: ^18^F-FDG-PET/CT fused images (**top row**) transaxial, coronal and sagittal view, PET images (**middle row**) and CT images (**bottom row**) where the lesion in the bladder wall is no longer visible.

**Figure 4:**
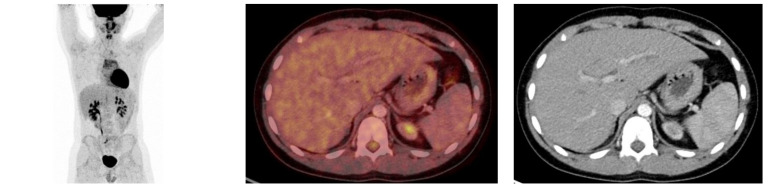
Follow-up: ^18^F-FDG-PET/CT maximal intensity projection (**left**), transaxial fused PET/CT image (**middle**) and CT image (**right**) of the liver where the previously seen lymph nodes in the liver hilum are no longer visible.

## References

[1] Bey E., Teklali Y., Rabattu P., Lapierre S.G., Piolat C. (2017). Case: Eosinophilic cystitis presenting as a bladder mass in an 11-year-old girl. Can. Urol. Assoc. J..

[2] Elmanzalawy A., Vali R., Chavhan G.B., Gupta A.A., Omarkhail Y., Amirabadi A., Shammas A. (2020). The impact of 18F-FDG PET on initial staging and therapy planning of pediatric soft-tissue sarcoma patients. Pediatr. Radiol..

[3] Van den Ouden D. (2000). Diagnosis and Management of Eosinophilic Cystitis. A Pooled Analysis of 135 Cases. Eur. Urol..

[4] Sparks S., Kaplan A., DeCambre M., Kaplan G., Holmes N. (2013). Eosinophilic cystitis in the pediatric population: A case series and review of the literature. J. Pediatr. Urol..

[5] Harris R.S., Venegas J.G., Wongviriyawong C., Winkler T., Kone M., Musch G., Melo M.F.V., de Prost N., Hamilos D.L., Afshar R. (2011). 18F-FDG Uptake Rate Is a Biomarker of Eosinophilic Inflammation and Airway Response in Asthma. J. Nucl. Med..

